# Characterisation of the koala (*Phascolarctos cinereus*) pouch microbiota in a captive population reveals a dysbiotic compositional profile associated with neonatal mortality

**DOI:** 10.1186/s40168-023-01527-9

**Published:** 2023-04-15

**Authors:** Toby I. Maidment, Emily R. Bryan, Michael Pyne, Michele Barnes, Sarah Eccleston, Samantha Cunningham, Emma Whitlock, Kelsie Redman, Vere Nicolson, Kenneth W. Beagley, Elise Pelzer

**Affiliations:** 1 Centre for Immunology and Infection Control, Queensland University of Technology, 300 Herston Rd, Brisbane, QLD 4001 Australia; 2 Currumbin Wildlife Hospital, 27 Millers Dr, Currumbin, QLD 4223 Australia; 3 Dreamworld Wildlife Foundation, Dreamworld Parkway, Coomera, QLD 4209 Australia; 4 Billabong Zoo Koala and Wildlife Park, 61 Billabong Drive, Port Macquarie, NSW 2444 Australia; 5 Paradise Country, Production Drive, Oxenford, QLD 4210 Australia

**Keywords:** Koala, Marsupial, Reproduction, Pouch, *Enterobacteriaceae*, Dysbiosis, Muribaculaceae, *Pluralibacter gergoviae*, *Klebsiella pneumoniae*, Endangered species, *Phascolarctos cinereus*

## Abstract

**Background:**

Captive koala breeding programmes are essential for long-term species management. However, breeding efficacy is frequently impacted by high neonatal mortality rates in otherwise healthy females. Loss of pouch young typically occurs during early lactation without prior complications during parturition and is often attributed to bacterial infection. While these infections are thought to originate from the maternal pouch, little is known about the microbial composition of koala pouches. As such, we characterised the koala pouch microbiome across the reproductive cycle and identified bacteria associated with mortality in a cohort of 39 captive animals housed at two facilities.

**Results:**

Using 16S rRNA gene amplicon sequencing, we observed significant changes in pouch bacterial composition and diversity between reproductive time points, with the lowest diversity observed following parturition (Shannon entropy — 2.46). Of the 39 koalas initially sampled, 17 were successfully bred, after which seven animals lost pouch young (overall mortality rate — 41.18%). Compared to successful breeder pouches, which were largely dominated by Muribaculaceae (phylum — *Bacteroidetes*), unsuccessful breeder pouches exhibited persistent *Enterobacteriaceae* (phylum — Proteobacteria) dominance from early lactation until mortality occurred. We identified two species, *Pluralibacter gergoviae* and *Klebsiella pneumoniae*, which were associated with poor reproductive outcomes. In vitro antibiotic susceptibility testing identified resistance in both isolates to several antibiotics commonly used in koalas, with the former being multidrug resistant.

**Conclusions:**

This study represents the first cultivation-independent characterisation of the koala pouch microbiota, and the first such investigation in marsupials associated with reproductive outcomes. Overall, our findings provide evidence that overgrowth of pathogenic organisms in the pouch during early development is associated with neonatal mortality in captive koalas. Our identification of previously unreported, multidrug resistant *P. gergoviae* strains linked to mortality also underscores the need for improved screening and monitoring procedures aimed at minimising neonatal mortality in future.

Video Abstract

**Supplementary Information:**

The online version contains supplementary material available at 10.1186/s40168-023-01527-9.

## Background

The koala (*Phascolarctos cinereus*) is an arboreal marsupial species endemic to the sclerophyll forests of eastern Australia, with a distribution range spanning from northern Queensland to South Australia. While southern koala populations (Victoria and South Australia) are considered stable, northern populations in Queensland, New South Wales, and the Australian Capital Territory are listed as endangered due to unsustainable levels of population decline [1–3]. The most significant contributor to this decline is widespread habitat loss, with car strikes, predation, extreme weather events, and diseases such as chlamydiosis and koala retrovirus (KoRV) also presenting significant sources of mortality [1, 4–8]. Additionally, increased fragmentation within core habitat zones has also reduced interpopulation gene flow, which presents a threat to long-term species viability due to loss of genetic diversity [9]. To stabilise koala populations and maintain genetic diversity, current management approaches are increasingly reliant on the establishment of an assurance population via captive breeding. These populations serve as a disease-free living genome bank of the species, which will be vital for future repopulation and translocation efforts. As such, ensuring high reproductive success amongst captive koalas is essential for overall species conservation.

Despite koalas having relatively high conception rates in captivity (approx. 80%), neonatal mortality (or ‘pouch death syndrome’) is a growing concern for breeding facilities, posing considerable limitations on the overall efficacy of breeding programmes [10, 11]. While several historical sources cite average mortality rates of 10–37%, the current incidence rate is unclear due to a lack of published data from the past 20 years [11–13]. Nevertheless, neonatal mortality remains an issue for several facilities in South-East Queensland, which have identified a group of otherwise healthy females who consistently fail to mature pouch young each season (personal communication – V. Nicolson, G. Tzipori, S. Eccleston). Mortality of pouch young in these individuals typically occurs during the first 7 months of neonatal development prior to emergence from the pouch (herein *early lactation*), despite a lack of complications during parturition [11, 12]. Notwithstanding idiopathic miscarriage, bacterial infections following exposure in the pouch are considered the most common cause of neonatal mortality, with several taxa identified as causative agents [11–13]. However, the environmental and host risk factors contributing to such infections remain poorly understood, leaving veterinarians with limited preventative screening or treatment options.

Like other marsupials, koalas give birth after a short gestation period of 34–36 days to immunologically naïve, physiologically undeveloped young, which continue their development ex utero in the marsupium (herein *pouch*), sustained via lactation [10, 14]. The maternal pouch is the primary site of immunological and physical development for marsupials, providing a humid, thermostable environment as well as protection from predation during lactation [14, 15]. However, the pouch is not sterile and has been demonstrated to harbour diverse communities of microorganisms including several pathogens, which may pose a risk to developing young [15–20]. Direct protection of neonates is provided through the transfer of immunoglobulins and antimicrobial peptides (AMPs) in the milk [21], along with epithelial secretions of broad-spectrum AMPs which regulate microbial growth in the pouch [15, 22]. These secretions may also facilitate compositional changes in the pouch microbiota towards a state beneficial for reproductive success.

Microbial communities in the marsupial pouch have been characterised in several species. In the tammar wallaby (*Macropus eugenii*) and Quokka (*Setonix brachyurus*), cultivation-based studies observed substantial reductions or complete clearance of culturable bacteria isolated from the pouches of mothers prior to and immediately following parturition [19, 20]. Similar reductions in microbial growth were observed in molecular-based studies of tammar wallaby and brushtail possum (*Trichosurus vulpecula*) pouches and were accompanied by major changes in bacterial composition around the time of birth [16, 18]. This was then explored further via 16S rRNA gene amplicon sequencing in the southern hairy-nosed wombat (*Lasiorhinus latifrons*) pouch, where microbial community composition and diversity dramatically changed according to host reproductive stage [23]. 16S rRNA gene amplicon sequencing of the Tasmanian devil pouch (*Sarcophilus harrisii*) also identified compositional differences between lactating and non-lactating females, though no significant difference in diversity was observed [24]. The koala pouch microbiota has not yet been characterised using cultivation-independent techniques; however, cultivation-dependent analysis by Osawa et al. (1992), identified similar patterns of microbial clearance prior to and following parturition [12]. Interestingly, this investigation also observed a lack of microbial clearance from the pouch following parturition in mothers who later lost young, suggesting dysregulation in the microbiota is associated with mortality [12]. While this highlights a possible cause of neonatal mortality, cultivation-independent analysis is required to determine whether these observations represent a difference in microbiota composition between successful and unsuccessful mothers.

It has long been known that microbial communities of the mammalian female reproductive system play important roles in reproductive and neonatal health. This has been demonstrated thoroughly in humans, where perturbations in various female reproductive tract-associated microbiota are linked to reduced fertility, miscarriage, and preterm birth, as well as increased susceptibility to several diseases [25–30]. Although comparatively less is known about the function of reproductive-associated microbiota in marsupials, similar perturbations in the pouch environment may also be associated with poor reproductive outcomes in koalas. This is particularly relevant in captive populations, as these perturbations could be influenced by environmental changes associated with captivity such as increased exposure to humans and artificial environments [31]. However, no studies to date have investigated the marsupial pouch microbiota in the context of developmental outcomes.

In this study, we employed both 16S rRNA gene amplicon sequencing and cultivation techniques to characterise bacterial communities in the koala pouch at various stages across the reproductive cycle, including where mortality occurred. From this data, we aimed to identify changes in community composition and diversity associated with reproductive status in healthy animals and to identify any community-wide changes or individual taxa associated with neonatal mortality. This was based on our hypothesis that neonatal mortality would be associated with a distinct microbial community profile in the pouch different to that of successful breeders. Through cultivation, we also aimed to establish a biobank of strains, which could be used for further characterisation of taxa linked to mortality, as well as future applications such as diagnostic development.

## Methods

### Ethics

All sample collection was performed with approval from the Queensland Government Department of Environment and Science (scientific purposes permit no. WA0023790) and Queensland University of Technology Animal Ethics Committee (approval no. 2000000165). All animals were handled by experienced veterinary professionals, and none was harmed at any point during sample collection.

### Animal husbandry and captive breeding

Sampling of koalas was carried out at two veterinary facilities — Currumbin Wildlife Hospital (CWH) and Dreamworld Wildlife Hospital (DW), both of which are located on the Gold Coast, Queensland, Australia.

CWH is a wildlife hospital and educational centre located in Currumbin, Queensland, Australia. Koalas at CWH are housed between two enclosures. The first enclosure, which is used for housing most female koalas and is located on hospital grounds, measures 11 × 15 m (165 m^2^ total area) and is contained by 1.2-m steel fencing. This enclosure contains natural dirt flooring, is surrounded by trees, and has no cover. The second enclosure, which is used to house one to three koalas at a time for tourism purposes, measures 11 × 10 m (110 m^2^ total area), and is contained by 1.2-m glass fences. This enclosure contains natural dirt flooring, is built into a human-occupied environment, and contains a mesh shade sail as covering. Poles and other inorganic surfaces in both enclosures are disinfected once per fortnight using F10 disinfectant at a 1:500 dilution. Females are housed separately from males.

Dreamworld Wildlife Foundation’s (DW) wildlife hospital and animal housing facility is situated at Coomera, Queensland, Australia. Koalas at DW were housed between six enclosures, all of which measure approx. 5 × 6 m (30 m^2^ total area) and house three to four koalas each. Enclosures are surrounded by loose steel fencing, have concrete floors and are sheltered, but are well ventilated. Poles, flooring, and inorganic surfaces are cleaned twice weekly using detergent. Females are housed separately from males.

As koalas are seasonal breeders, mating in captivity is initiated each year between September and November. To initiate mating, breeding-age dams displaying behavioural oestrus are coupled with a mature sire by temporarily introducing them to the male enclosure, with activity monitored by veterinary staff. Once copulation has occurred, the dam is then transported back to the female enclosure and monitored until gestation is complete or rebreeding is required.

Koalas at both facilities are monitored by veterinary staff regularly throughout the day and fed various Eucalyptus cuttings sourced from local plantations ad libitum. Following breeding, developmental progress of young is monitored through regular pouch-checking by veterinary staff prior to emergence and monthly weighing once fully furred to monitor nutritional status.

### Study design

Female koalas give birth to one ~ 0.5-g offspring after 34–36-day gestation, which subsequentially climbs from the urogenital opening and into the pouch and continues development over the following 12 months until weaning and dispersal [10]. Koala development can be separated into three distinct phases based on milk composition and behaviour: early lactation, emergence/pap feeding, and late lactation.

**Fig. 1:**
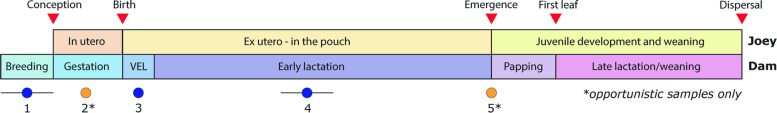
Timeline displaying sample collection relative to neonatal developmental phases and reproductive cycle. Entire timeline is representative of 12 months, with major developmental events displayed above marked by red arrows and sampling time points marked with circles below. Opportunist time point samples, noted in orange and with an asterisk, were not collected in all longitudinally sampled dams

To investigate how the pouch microbiota changes with host reproductive status, collection of pouch samples was carried out at intervals corresponding with distinct reproductive/developmental phases, as detailed in Fig. 1. Major time points were collected at both facilities and included anoestrus (prior to behavioural displays of oestrus and copulation); very-early lactation (VEL; < 4 days of parturition); and early lactation (EL; approx. 1–3-month post-partum). Minor time points, which were collected opportunistically where suitable, included oestrus/cycling (2 weeks post copulation) and post-emergence (PE; ~ 6–8-month post-partum following emergence from the pouch). Wherein which mortality of pouch young occurred, samples were also collected immediately following notification of veterinarians.

### Sample collection

Samples were collected from koala pouches at each time point using two types of collection swabs. The first was collected using a COPAN regular FLOQ® swab (cat. no. 552C; COPAN, CA, USA) and used for amplicon sequencing, while the second was taken collected using a COPAN regular ESwab® containing 1-mL liquid amies media (cat no. 4E039S; COPAN, CA, USA) for cultivation.

**Fig. 2:**
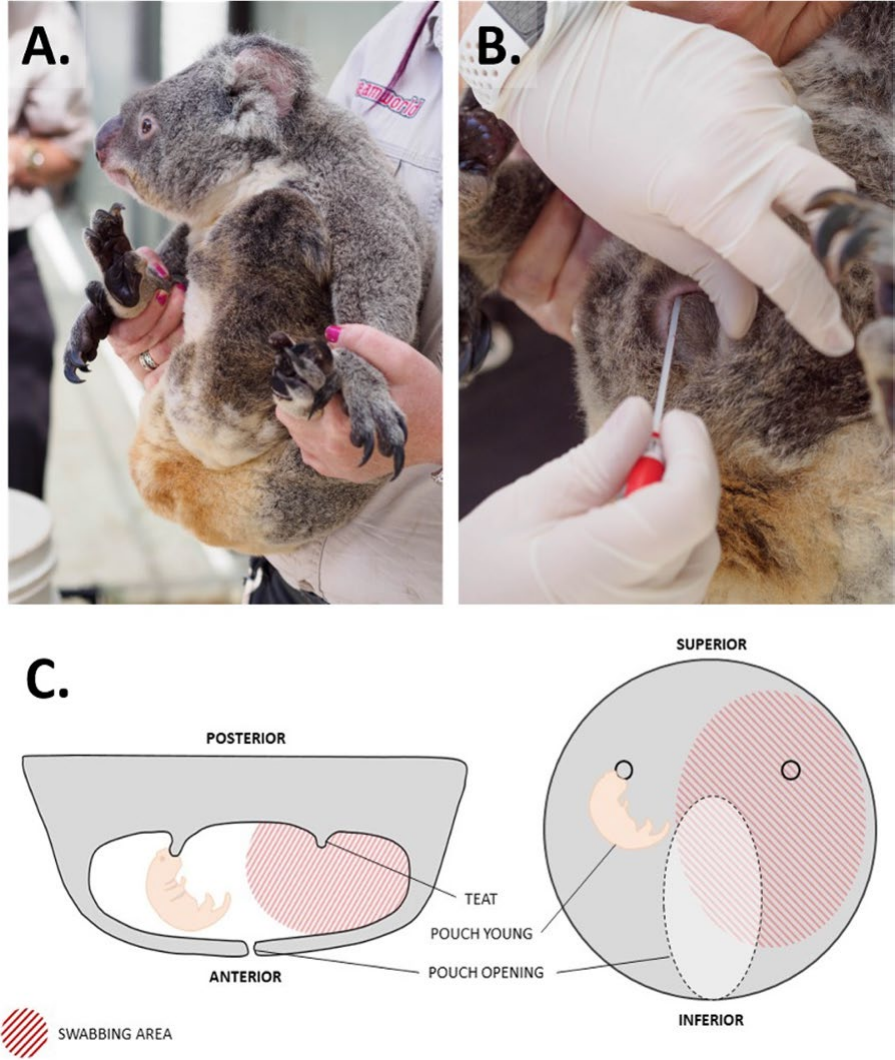
Swab collection from koala pouches. **A** Prior to sample collection, koalas are gently restrained by trained personnel using the fore and hindlimbs to prevent injury. **B** To collect samples, the pouch is propped open using hair on the anterior edge of the pouch, and the swab is carefully inserted into the pouch perpendicular to the unoccupied teat. Samples were collected from the area displayed in (**C**)

To collect swab samples, koalas were restrained by trained veterinary personnel, and their pouch opened using hair on the anterior edge of the pouch opening as seen in Fig. 2 A–B. Ensuring no contact with outside surfaces, the swab was then gently inserted into the pouch perpendicular to the vacant teat and rotated over the epithelial pouch lining with moderate pressure for approximately 30 s (Fig. 2C). Veterinary staff were required to wear sterile nitrile gloves during sample collection and wash their hands with 2% chlorhexidine hand-wash beforehand to minimise contamination. Caution was taken to ensure swab tips only contacted the inner pouch surface to minimise contamination from skin microorganisms, with veterinary staff instructed to repeat any swabs that may have touched unwanted locations.

Using the same swab types, environmental control samples were also collected to monitor for exogenous contamination of the pouch from environmental reservoirs during both sequencing and cultivation. Environmental negatives were collected during each sampling round and were collected by uncapping and waving swabs in the air for 30 s. Positive environmental control samples were also collected from *Eucalyptus* feed, poles, and surfaces in direct contact with koalas at several time points.

Swab samples used for sequencing were stored at − 80 °C immediately following collection, while swabs for cultivation were stored at 4 °C following collection. All samples were transported to our laboratory facility in Brisbane, Queensland, Australia, on wet ice within 72 h of collection and stored/processed immediately.

### DNA extraction and 16S rRNA amplicon sequencing

Swab tips were transferred to sterile 1.5-mL tubes containing 180-μL lysis buffer and incubated at 37 °C for 60 min with regular vortex agitation. The lysis buffer contained 20 mg/mL lysozyme (Sigma, Australia; cat. no. L4919-1G; lot no. SLCC4285), 20 U/mL lysostaphin (Sigma-Aldrich, Germany; cat. no. L386-5MG; lot no. 079M4019V), 20-mM Tris–HCl, 2-mM EDTA, and 1.2% Triton® X-100 (Merck, Darmstadt, Germany; cat. no. 9036–19-5) in molecular-grade double-distilled H_2_O (cat no. 10977015; Invitrogen, Australia) at pH 8.0 and was filter-sterilised using a 0.2-µM membrane filter prior to use.

Following enzymatic lysis, DNA was extracted using the QIAmp® DNeasy PowerLyzer PowerSoil Kit (cat. no. 12855–100; QIAGEN, Germany) according to the manufacturer’s protocol, with DNA eluted in 50-μL molecular-grade ddH2O (cat. no. 10977015; Invitrogen, Australia) and sample homogenisation performed using a Biospec Mini-BeadBeater 16 (Biospec, Oklahoma, USA). DNA samples were stored at − 20 °C until further processing.

### Negative and positive sequencing controls

Techniques used for amplification and sequencing the 16 s rRNA gene are highly susceptible to contamination, both in the field from environmental contaminants and user-end contamination during sample processing. As such, in line with recommendations made by Eisenhofer et al. (2019), our study included several control samples to account for such issues [32]. These included environmental negative controls (sample blanks), water-only controls, and extraction-negative controls (pooled). Collectively, these controls enable the monitoring of contaminants in the collection environment, packaged swabs, elution water, and kits/reagents.

Bacterial-positive controls were also used during sequencing to inspect taxonomic coverage and any biases that may skew representation therein as suggested. The bacterial organisms used for constructing positive control communities were chosen based on previous microbial investigations of marsupial pouches and phylogenetic coverage. These organisms are listed in Table 1 and were used to make two positive control mock communities: the first (POS-MIX) containing 20 ng/µL each of 5 × mixed organisms and the second (POS-ENT) containing 25 ng/µL of 4 × gram-negative bacilli. POS-MIX was used to inspect for biases amongst a wide range of bacteria which may be present in the pouch environment, while POS-ENT was used to assess the efficacy of genus-level resolution within the family Enterobacteriaceae, which contains several taxa previously implicated with neonatal mortality in captive koalas [11, 12].

**Table 1:** Positive control organisms and their corresponding pooled sequencing sample

Organism and strain number	Positive control sample
*Staphylococcus aureus* NCTC6571	POS-MIX
*Enterococcus faecalis* ATCC29212	POS-MIX
*Serratia marcescens* ATCC14756	POS-MIX
*Escherichia coli* ATCC8739	POS-MIX
*Corynebacterium diphtheriae* NCTC3529	POS-MIX
*Klebsiella pneumoniae* ATCC13883	POS-ENT
*Pluralibacter gergoviae* ATCC33028	POS-ENT
*Enterobacter cloacae* ATCC13047	POS-ENT
*Citrobacter freundiii* ATCC14135	POS-ENT

For each of these organisms, fresh 1:100 dilutions of overnight cultures were made up in 1-mL brain–heart infusion (BHI) broth and incubated at 37 °C to an optical density at 600 nm (OD600) of 0.6. Cultures were then pelleted via centrifugation at 500 × g for 10 min (7500 RPM), resuspended in 180-μL lysis buffer, and processed using the DNA extraction protocol used for swab samples. DNA quality and concentration were performed using a Qubit fluorometer (cat no. Q32851; Invitrogen, Australia), and mock communities were made up to a final concentration of 100 ng/µL in 100 µL molecular-grade ddH_2_O as stated above.

### 16S rRNA amplicon sequencing and bacterial community profiling

Library preparation and 16S rRNA gene amplicon sequencing of sample DNA was performed at the Australian Genomics Research Facility (Melbourne, Victoria, Australia) on the Illumina MiSeq platform (2 × 300 bp chemistry) using universal primers 341F (5′-CCTACGGGNGGCWGCAG-3’′) and 806R (5′-GGACTACHVGGGTWTCTAAT-3′) targeting the V3-V4 hypervariable region. These primers were chosen based on in silico predictions of database coverage and were validated in vitro via qPCR using swab sample DNA and bacterial positive control organisms.

Following sequencing, demultiplexed, trimmed, 300-bp paired-end reads were imported into *Quantitative Insights into Microbial Ecology-2* (QIIME2; v2021.4) [33]. In brief, reads were screened for adapter sequences using *Cutadapt* and quality checked with Q2-*Demux*, with denoising and amplicon sequence variant (ASV) assignment performed on quality-filtered reads using the *Deblur* ‘denoise-16S’ [34] tool at a trimmed length of 266 bp. Rooted and unrooted phylogenetic trees were then generated using *align-to-tree-mafft-fasttree* [35, 36].

Representative sequences were then assigned taxonomy using both a region-specific classifier with SILVA taxonomy (SSUr138 NR_99; https://www.arb-silva.de/) [37], as well as a full-length classifier based on the National Centre for Biomedical Innovation (NCBI) 16S rRNA gene database (NCBI BioProjects 33,175 and 33,317). Both taxonomic classifiers were built using *q2-rescript* [38] and trained using the *fit-classifier-naïve-bayes* tool prior to use. Classification was performed using *Classify-sklearn* [39], with output taxonomic files merged using *q2-rescript* ‘merge-taxa’, and inconsistent taxonomic handles resolved using the R package phyloseq (v3.14.1) [40]. Feature tables were filtered to remove rare taxa (present in < 2 samples), taxa that could not be assigned past the domain level and reads originating from chloroplasts and mitochondria. The resulting taxonomic data was then used to screen for putative contaminants with the R package *decontam* [41], with suspected contaminant taxa identified based on their prevalence in negative controls (threshold = 0.1) and removed where appropriate. Taxonomic analysis and visualisation were performed on relative abundance tables filtered as necessary using QIIME2 and *ggplot2* [42].

Alpha- and beta-diversity calculations were generated using the *q2-diversity core-metrics phylogenetic* tool from a feature table rarefied at a depth of 1000 reads per sample. Alpha-diversity metrics included Shannon’s richness (or entropy) and observed species indices, with statistically significant differences between groups identified using the Kruskal–Wallis test via the *q2-diversity alpha-group-significance* tool [43]. For beta diversity, weighted UniFrac distances from rarefied data was used for ordination via principal coordinates analysis (PCoA), with statistically significant differences between groups identified using analysis of similarity (ANOSIM) testing with 4000 permutations.

Differential abundance testing was performed with Deseq2 [44] using the geometric means of CLR-transformed count data. Implementation of the Wald test with Benjamini–Hochberg multiple test corrections was used to identify significantly differentially abundant taxa between conditions, with an FDR-adjusted *P*-value (p-adj) < 0.05 deemed statistically significant.

### Cultivation of microorganisms from swab samples

In addition to bacterial community profiling, bacteria were cultivated and isolated from pouch swab samples to establish a biobank of strains for downstream use. In brief, 100-mL fresh ESwab sample eluent from each sample was inoculated onto one nutrient agar (NA; cat. no. CM0003B; Oxoid, Australia) and two brain–heart infusion (BHI) plates (cat. no. CM11135B/LP0011; Oxoid, Australia) then incubated at 37 °C for 24 h (O_2_ for 1 × BHI and 1 × NA; 5% CO_2_ for 1 × BHI). Macroscopically distinct bacteria were then further isolated by inoculating onto fresh agar and incubating under previously successful conditions for a further 24 h. All isolated organisms were then gram-stained for rudimentary identification and frozen at − 80 °C in 500-µL BHI broth (cat. no. CM11135B; Oxoid, Australia) with 50% glycerol (0.22 µM filter-sterilised) until further processing.

To control for environmental contamination, sample collection negatives from each sampling event/date were cultured alongside animal samples. Experimental contamination of samples was also controlled for through by incubating a BHI plate open in the Biosafety Cabinet-II throughout the inoculation process.

### Cultivation-based identification and disk diffusion antibiotic susceptibility testing of Pluralibacter gergoviae and Klebsiella pneumoniae recovered from pouch samples

To characterise *P. gergoviae* and *K. pneumoniae* present in koala pouches during loss, all loss-associated organisms which presented as gram-negative bacilli and exhibited both aerobic and anaerobic growth were revived by cultivation on nutrient agar for 24 h at 37 °C O_2_. Based on 16 s rRNA amplicon sequencing results, suspected *P. gergoviae* isolates were also cultivated from culture-positive healthy animal samples using the same criteria. Following revival, isolates were gram-stained again to confirm microscopic morphology, replated onto BHI agar, and incubated for a further 18 h at 37 °C O_2_.

To provide a preliminary identity, isolates were tested using analytical profile index (API) 20-E (bioMerieux, France; cat. no. 09567D; lot no. 1006810990) testing strips as per manufacturers’ guidelines, with *P. gergoviae* (ATCC33028), *Escherichia coli* (ATCC8739), and *K. pneumoniae* (ATCC13883) used as calibration standards. In addition to API testing, isolates were also subjected to rapid oxidase testing (Becton Dickinson; cat. no. C561A01; lot no. 2478–0020-417) and were grown on MacConkey (Oxoid, UK; cat. no. CM0115; lot no. 1665120) agar to assess lactose fermentation. All isolates identified as *P. gergoviae* or *K. pneumoniae* were then used in subsequent procedures.

Antibiotic susceptibility testing was performed via disk diffusion using the CDS method [45]. Testing was performed on nine pouch isolates (eight *P. gergoviae*, one *K. pneumoniae*), with *P. gergoviae* (ATCC33028) and *K. pneumoniae* (ATCC13883) used as controls. Prior to commencement, bacteria were grown in pure culture on nutrient agar for 18 h at 37 °C O_2_. Colonies were then suspended in 6-mL sterile saline (0.95% NaCl) until turbidity was equal to that of an 0.5 MacFarland standard (ThermoFisher Scientific, Victoria, Australia; cat. no. E1041) and inoculated onto two SensiTest™ agar plates (ThermoFisher Scientific, Australia; cat. no. PP2017; lot no. 4321850) each using a sterile cotton swab. After drying for 5 min, four antibiotic disks were applied to each plate. Plates were then incubated 18 h at 37 °C O_2_, after which zones of inhibition were measured and antibacterial susceptibility confirmed according to the CDS database for *Enterobacteriales* [45].

Antibiotics used were 25-µg ampicillin (cat. no. CT0004B; Oxoid, Australia), 10-µg tetracycline (cat. no. CT0053B; Oxoid, Australia), 2.5-µg ciprofloxacin (cat. no. CT1136S; Oxoid, Australia), 10-µg gentamycin (cat. no. CT0024B; Oxoid, Australia), 10-µg ceftazidime (lot no. 3320388; Oxoid, Australia), 15-µg azithromycin (cat. no. CT0906B; Oxoid, Australia), 30-µg chloramphenicol (cat. no. CT0013B; Oxoid, Australia), and 20-µg augmentin (cat. no. CT1510S; Oxoid, Australia). These antibiotics were chosen due to their use in the treatment of koalas and coverage of multiple drug classes.

## Results

### Sampling and breeding outcomes

A total of 39 koalas were sampled across both facilities during the 2020–2021 breeding season (July 2020–July 2021). Of the 12 animals initially sampled at CWH during anoestrus (time point 1), one animal was deemed too immature to breed, two mated unsuccessfully, and nine successfully gave birth to young without complications. At DW, 8 out of 27 animals sampled during anoestrus were bred during the sampling period, all of which successfully gave birth without complications. Four additional animals from DW were also captured at various other reproductive time points; however, longitudinal sampling could not be achieved for these animals due to breeding occurring outside of the experimental timeframe.

Regarding developmental outcomes, six out of nine joeys were lost at CWH and one out of 8 at DW during the sampling period. This brought mortality rates for the 2020–2021 breeding season to 66.66% at CWH and 12.5% at DW (41.18% across both facilities). It should also be noted that an additional three joeys were lost at DW in late 2020; however, these were not represented in our mortality rates as breeding of these animals occurred as part of the 2019–2020 season.

Of the seven young that passed away, four were found deceased in the pouch (animal IDs: CS-K1, CS-K2, CS-K5, CS-K12), and two were removed from the pouch due to ill health and died while receiving medical care (CS-K4, DW-K8; Table 2). All pouch young passed away ≤ 8-month *post-partum,* and except for CS-K5, all deaths occurred prior to papping. Following loss of young, one koala at CWH (CS-K2) was also found raising another dam’s joey (CS-K10) — the circumstances of which are unclear; however, this joey passed away at 10 months of age (Table 2). Causes of death for all losses remained unknown at the time of writing, and only one loss (CS-K1) occurred in an individual with a history of neonatal mortality.

**Table 2:** Details of joey losses occurring during the study

Dam ID	Facility	Age at time of death	Circumstance of death
CS-K1	Currumbin	6 months, 16 days	Found deceased in pouch during routine check. Undetermined
CS-K2	Currumbin	4 months, 25 days	Found deceased in pouch during routine check. Undetermined
CS-K4	Currumbin	7 months, 22 days	Deceased after removal from pouch due to ill health. Undetermined
CS-K5	Currumbin	8 months	Found deceased in pouch. Otherwise healthy and able to move outside the pouch. Suspected head trauma
CS-K12	Currumbin	5 months, 27 days	Found deceased in pouch during routine check. Undetermined
DW-K8	Dreamworld	6 months, 3 days	Removed from pouch due to ill health and low weight. Passed away 2 weeks later. Undetermined

### 16S rRNA gene amplicon sequencing and cultivation data overview

A total of 93 pouch swab samples were collected in duplicate from 39 koalas, of which 17 were bred and captured longitudinally (*CWH* = 9, *DW* = 8). Stratified by reproductive time point, these included 38 anoestrus/pre-breeding samples, seven oestrus/gestation samples (collected opportunistically), 20 very-early lactation samples, 16 early lactation samples, six postemergence samples, and six post mortality samples.

Sequencing was performed on 115 samples in total (93 biological, 22 controls; Supplementary Table 1), which yielded a total of 10,146,779 forward and reverse reads. Following denoising and ASV assignment using *Deblur*, 7734 features were identified across the 114 samples passing quality control, with a total frequency of 1,649,153. Using a prevalence-based decontamination threshold of 0.1, we identified 20 putative contaminants, of which 10 were removed (1 × *Ralstonia* sp., 1 × *Pelomonas*, 3 × *Streptococcus* sp., and 5 × *Staphylococcus* sp.). The remaining 10 taxa were not removed, as they were identified as contaminants based on negative control samples sharing near-identical composition to neighbouring, high-biomass, positive control samples.

In addition to culture-independent profiling, all pouch swabs from the 17 animals sampled longitudinally were cultivated in vitro to obtain bacterial isolates for downstream use. All negative control samples were also cultivated to control for contamination. Through this process, we established a biobank of 235 unique bacterial isolates which were gram stained for rudimentary identification and stored at − 80 °C until further use (see supplementary Table 2). No bacteria were cultivated from sampling negative control samples.

### Pouch microbiota diversity changes with host reproductive status

As a potential adaptation to ex utero reproduction, the marsupial pouch environment typically undergoes significant physiological changes across the reproductive cycle. To examine how these changes impact on microbiota diversity in koalas, we first assessed the effect of reproductive status on community diversity.

**Fig. 3:**
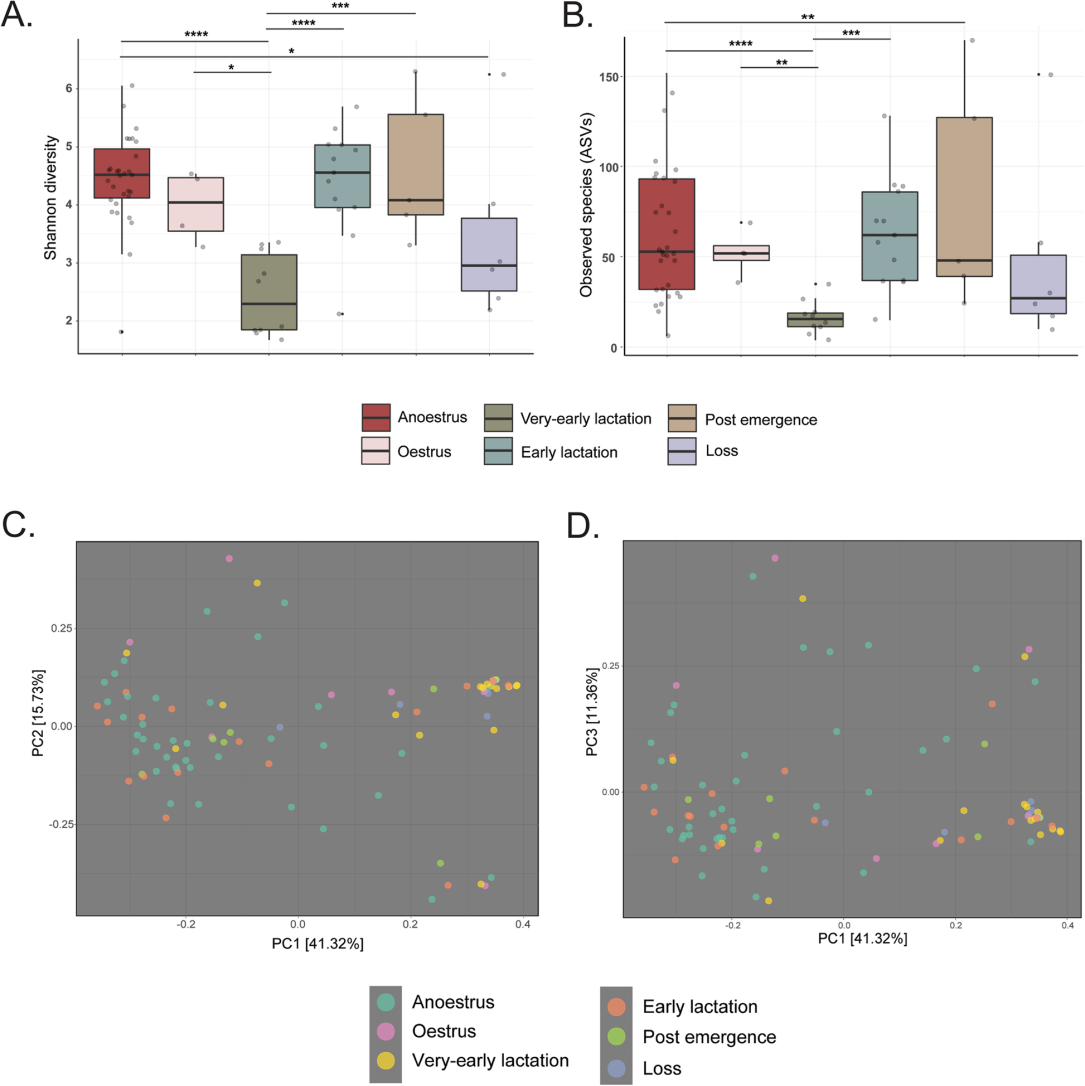
Bacterial community diversity in the pouch microbiota of captive koalas. Alpha- and beta-diversity metrics were calculated on a feature table rarefied to 1000 reads per sample. Alpha-diversity metrics included **A** Shannon diversity and **B** observed species (or ASVs), with Kruskal–Wallis testing performed to identify significant differences between groups. Beta diversity is presented as weighted UniFrac distances, with ordination performed via principal coordinates analysis (PCoA). **C** Displays ordination on a 2-dimensional PCoA plot with PC1 and PC2, while **D** displays data plotted to PC1 and PC3. *P < 0.05; **P < 0.01; ***P < 0.001; ****P < 0.00001

Regarding alpha-diversity metrics of Shannon diversity and observed ASVs, we observed significant variance in the pouch microbiota across the reproductive cycle (Shannon Kruskal–Wallis *P* < 0.001, *H* = 25.23; observed features *P* < 0.001, *H* = 21.03). In particular, pouch samples obtained following parturition (VEL; Shannon = 2.46; ~ 14 ASVs) exhibited significantly lower community richness (Kruskal–Wallis *P* < 0.005) than those collected during anoestrus (Shannon diversity = 4.23; ~ 55 ASVs), oestrus (Shannon diversity = 3.03; ~ 38 ASVs), early lactation (Shannon diversity = 4.08; ~ 66 ASVs), and post emergence (Shannon diversity = 4.34; ~ 77 ASVs). Samples collected following mortality of pouch young (Shannon diversity = 3.50; ~ 45 ASVs) also showed notably lower community richness compared to other samples, but only significantly differed from anoestrus samples, as detailed in Fig. 3 (Kruskal–Wallis *P* < 0.05). No significant differences in community richness were observed between facilities or breeding groups, irrespective of stratification by reproductive time point (Kruskal–Wallis *P* > 0.05).

Next, we performed ordination of weighted UniFrac distances via principal coordinates analysis (PCoA) to identify structural changes in the pouch microbiota across the reproductive cycle. As seen in Fig. 3 C–D, pouch samples visually separated across PC1 (accounting for 41.32% total variation) into two loose clusters — one containing anoestrus, early lactation, and postemergence samples and the other containing very-early lactation, loss, and oestrus samples (Fig. 3 C–D). This pattern was explained by compositional similarity within both clusters (ANOSIM *P* > 0.05) and significant compositional dissimilarities between groups driving separation across PC1 (ANOSIM *P* < 0.05). Demonstrating this, microbial composition in very-early lactation samples differed significantly to anoestrus (ANOSIM *P* < 0.0005; *R* = 0.357) and early lactation (ANOSIM *P* < 0.0005; *R* = 0.357) samples, but not oestrus or loss samples. Similarly, oestrus sample composition also differed significantly from anoestrus (ANOSIM *P* < 0.05; *R* = 0.253), while loss samples differed from both anoestrus (ANOSIM *P* < 0.05; *R* = 0.248) and postemergence samples (ANOSIM *P* < 0.05; *R* = 0.34).

**Fig. 4:**
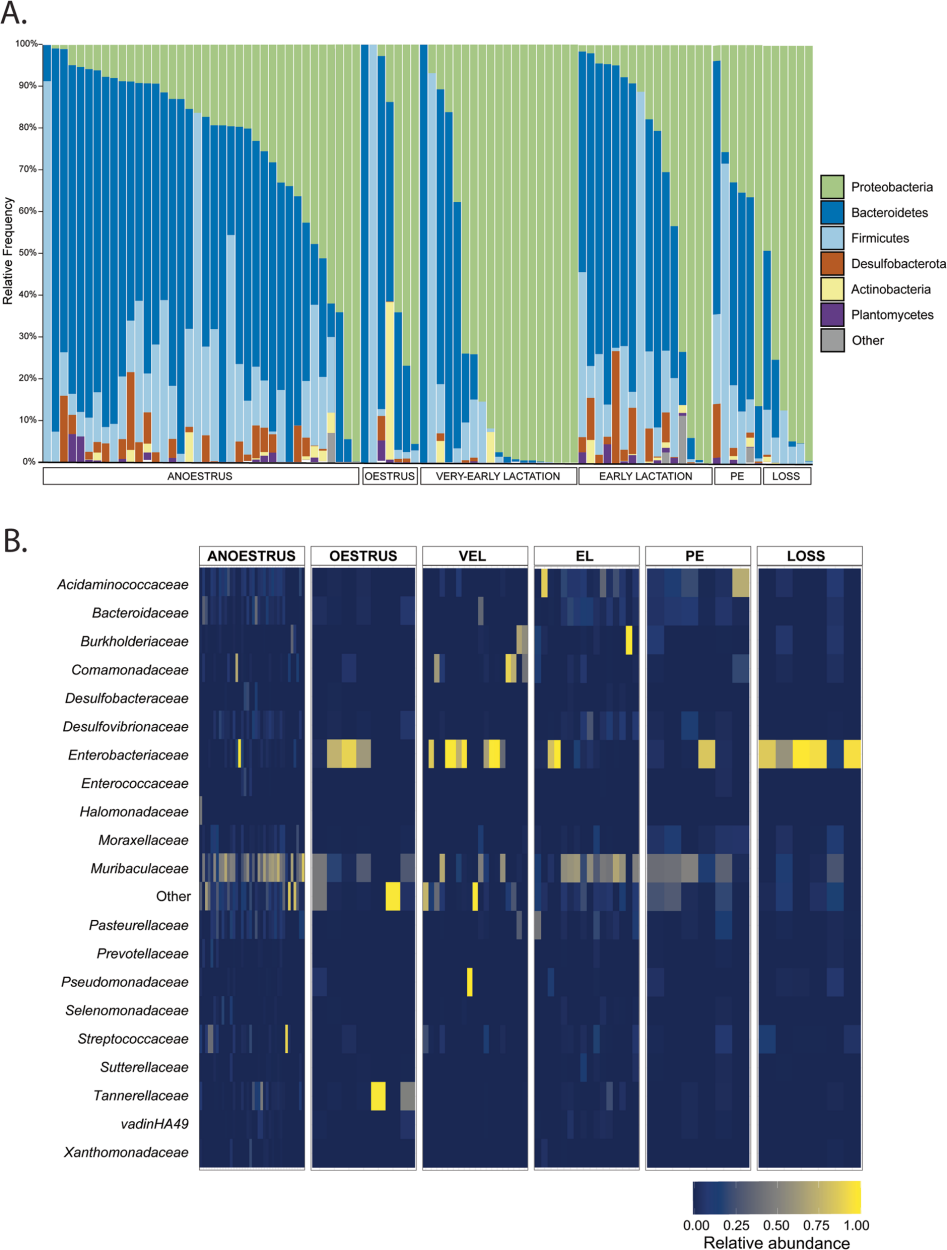
Phylum- and family-level bacterial composition in the pouch microbiota of captive koalas. The figure displays **A** phylum-level and **B** family-level bacterial composition in individual pouch samples grouped by reproductive time point. Values on both plots represent relative abundances of taxa. **A** Visualised using QIIME2 (v2021.4). **B** Visualised using ggplot2. VEL, very-early lactation; EL, early lactation; PE, post emergence

Similar to our observations regarding diversity and richness, taxonomic composition during very-early lactation was highly dissimilar to other reproductive time points. For instance, anoestrus, early lactation, and postemergence samples exhibited similar abundances of Proteobacteria (38.4–48.54%), *Bacteroidetes* (35.74–44.48%), *Firmicutes* (10.22–12.53%), and Desulfobacteria (2.94–4.44%) at the phylum level, and Muribaculaceae, Enterobacteriaceae, and Acidaminococcaceae at the family level (Fig. 4). Oestrus samples also exhibited a similar taxonomic composition, comprising mostly of the phyla Proteobacteria (49.32%), *Bacteroidetes* (41.94%), and *Actinobacteria* (3.57%), with the most abundant families being Enterobacteriaceae (46.51%), Muribaculaceae (26.73%), and Tannerellaceae (11.82%).

In contrast, very-early lactation samples were highly dominated by Proteobacteria (90.70%), contained low abundances of *Bacteroidetes* (5.67%) and *Firmicutes* (3.29%), and were represented primarily by the families Enterobacteriaceae (69.23%), and Pseudomonadaceae (17.8%), with low abundances of Muribaculaceae (5%) (Fig. 4; SI Tables 3 and 4). A similar compositional profile was also observed in samples collected following mortality. These samples primarily comprised of the phyla Proteobacteria (89.94%), along with *Bacteroidetes* (5.67%) and *Firmicutes* (4.15%), with the most abundant families being Enterobacteriaceae (84.39%), Muribaculaceae (3.92%), and Streptococcaceae (2.24%; Fig. 4) (see supplementary Tables 5, 6, 7 and 8). It is important to note however that a high level of inter-individual differences in taxonomic composition were identified at each reproductive time point, as illustrated in Fig. 4A.

### Compositional differences in pouch microbiota between successful and unsuccessful breeders prior to loss of young

**Fig. 5:**
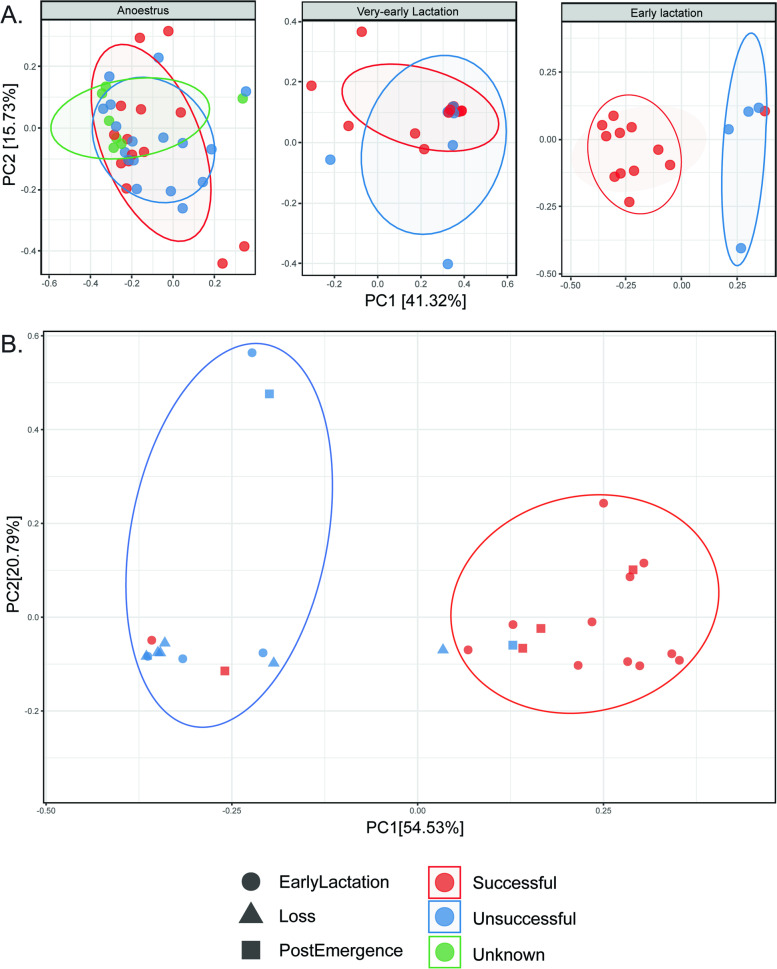
Bacterial community beta diversity stratified by breeding status. Ordination and principal coordinates analysis (PCoA) was performed using weighted UniFrac distances calculated on data rarefied to 1000 reads per sample. **A** Displays PCoA ordination of pouch samples faceted by time point at anoestrus, very-early lactation, and early lactation. **B** Displays ordination of early lactation, loss, and postemergence samples only using PC1 and PC2 axes

In addition to changes in the microbiota associated with maternal reproductive status, we also found that pouch microbiota composition differed notably between koalas who successfully reared young (successful breeders) and koalas who lost young during lactation (unsuccessful breeders). Firstly, we observed that while significant compositional shifts between anoestrus and very-early lactation occurred in both successful (ANOSIM *P* < 0.01; *R* = 0.258) and unsuccessful breeders (ANOSIM *P* < 0.01; *R* = 0.470), the trajectory of structural change between VEL and EL differed between groups (Fig. 5). In successful breeders, we observed significant separation between VEL and EL sample clusters across PC1 (ANOSIM *P* < 0.001; *R* = 0.458), whereas VEL and EL samples from unsuccessful breeders remained compositionally similar (ANOSIM *P* > 0.05; *R* =  − 0.042). This trend was then observed at subsequent time points (Fig. 5), with EL and loss samples clustering tightly in unsuccessful breeders (ANOSIM *P* > 0.05; *R* = 0.087) and EL samples clustering with PE samples in successful breeders (ANOSIM *P* > 0.05; *R* = 0.049).

**Fig. 6:**
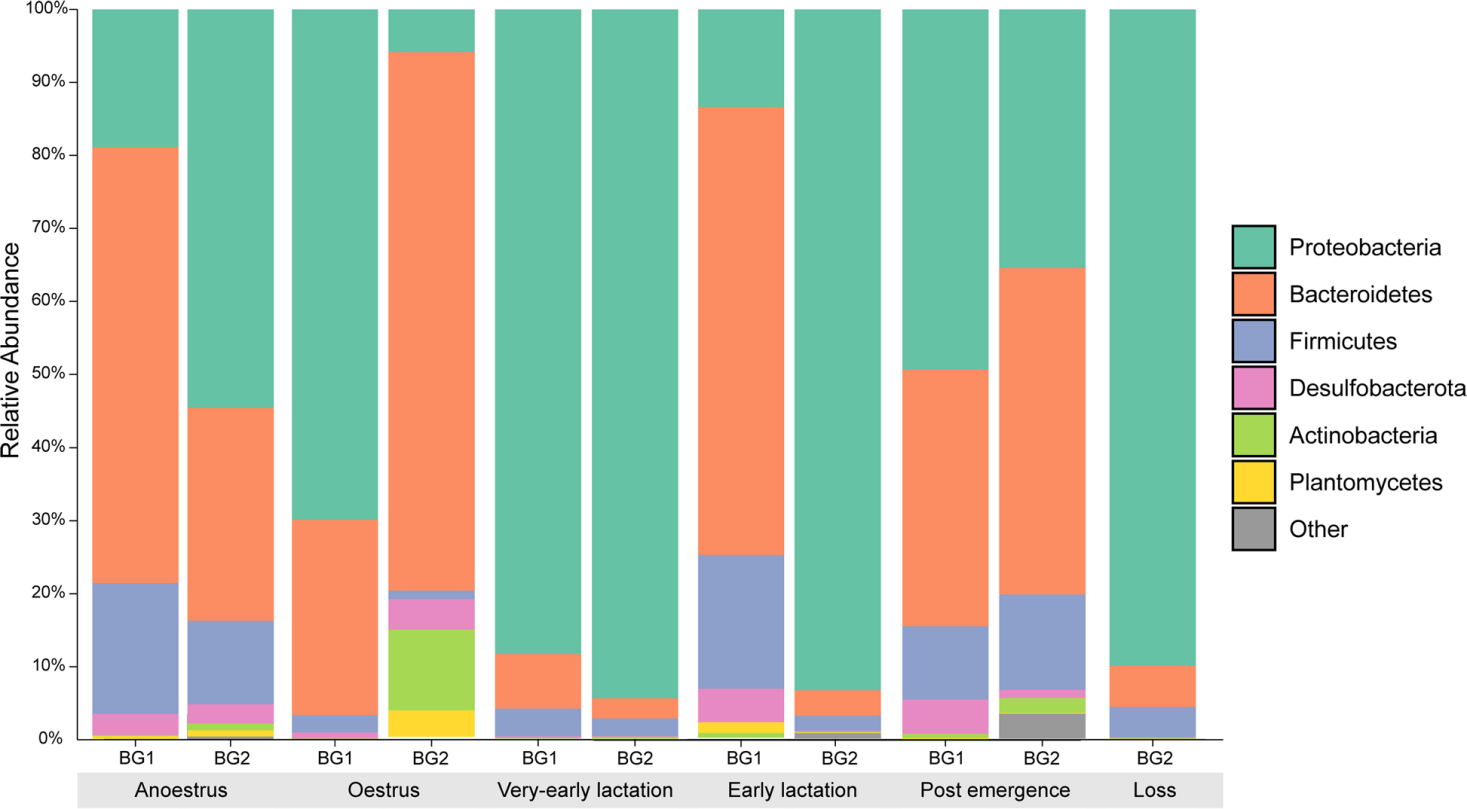
Phylum-level composition of the pouch microbiome at each reproductive time point grouped by breeding status. Samples were split by breeding group, where BG1 = successful breeders and BG2 = unsuccessful breeders. Plot was produced using QIIME2 (v2021.4)

We also observed several distinct differences in taxonomic composition between successful and unsuccessful breeders (Fig. 6). During anoestrus, successful breeder pouches were dominated by *Bacteroidetes* (59.68%), followed by Proteobacteria (18.94%), with the most abundant families being Muribaculaceae (51.65%), Pasteurellaceae (7.4%), Acidaminococcaceae (6.19%), and Moraxellaceae (4.06%). Meanwhile Proteobacteria represented the most abundant phylum in unsuccessful breeder pouches (54.65%), followed by *Bacteroidetes* (29.16%), with Enterobacteriaceae (39.89%), Muribaculaceae (17.8%), Tannerellaceae (5.13%), and Streptococcaceae (4.4%) representing the most abundant families. Interestingly, the most abundant taxon in unsuccessful breeder anoestrus samples, belonging to the genus *Pluralibacter* (38.87%), only accounted for 0.01% average relative abundance in successful breeder pouches during anoestrus (see Supplementary Tables 5, 6, 7, 8, 9 and 10).

During VEL, both breeding groups were dominated at phylum level by Proteobacteria. However, while Proteobacteria was represented in successful breeder samples by the families Enterobacteriaceae (54.91%) and Pseudomonadaceae (28.93%), unsuccessful breeder samples were instead dominated by *Enterobacteriaceae* (92.28%). The summed abundance of *Bacteroidetes* (BG1 — 7.46%; BG2 — 2.77%) was also lower in unsuccessful breeder VEL samples compared to those from successful breeders, which was reflected primarily by differing abundances of the family Muribaculaceae (BG1 — 6.41%; BG2 — 2.78%). *Pluralibacter* represented the most abundant genus in both groups during VEL (BG1 — 45.06%; BG2 — 59.27%), with other genera present including *Pseudomonas* (BG1 — 28.93%), *Escherichia-Shigella* (BG1 — 9.85%; BG2 — 33%), and *Muribaculum* (BG1 — 6.41%; BG2 —2.78%) (see Supplementary Tables 9 and 10).

**Fig. 7:**
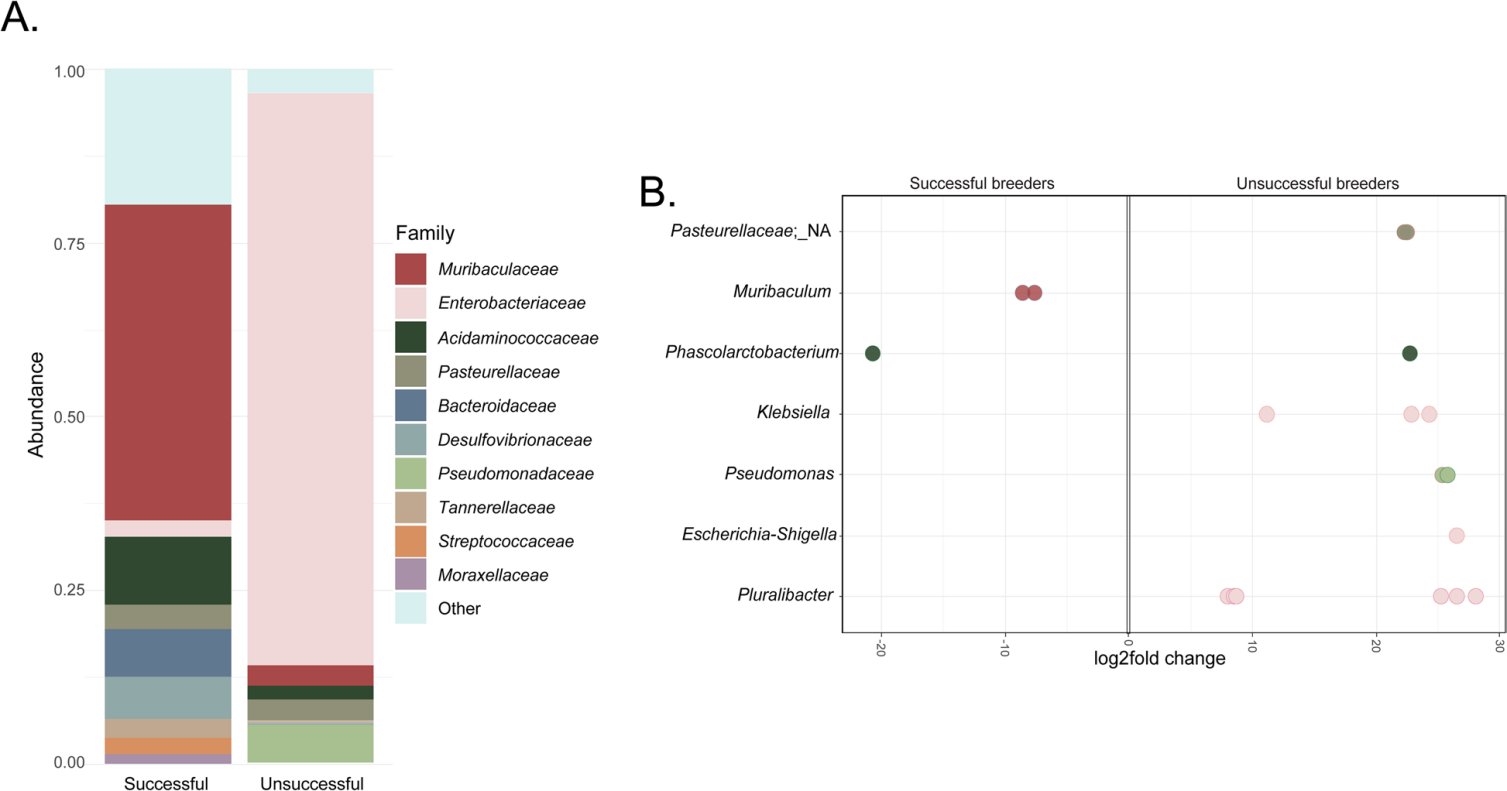
Family-level differences and differentially abundant genera in the pouch microbiota of successful vs. unsuccessful breeding koalas during early lactation. **A** Displays relative abundance bar plot of EL samples grouped by breeding status at the family level. **B** Differentially abundant ASVs (FDR-adj *p* < 0.05) in successful and unsuccessful breeder EL samples were identified using pairwise analysis in *DeSeq2* (v3.14). ASVs were assigned taxonomy at the genus (*y*-axis) and family (as per bar plot legend) level, with each point representing individual ASVs within genera. The *x*-axis shows the ‘log2 fold change’ in taxa between groups, with negative values representing higher abundance in successful breeders and positive values representing higher abundance in unsuccessful breeders. Visualisation was performed using QIIME2 (v2021.4) and *ggplot2* (v3.3.5). Raw data presented in Supplementary Tables 5, 6, 7, 8, 9, 10 and 11

As inferred by diversity results, the most notable difference in taxonomic composition between breeding groups was observed during EL (Fig. 7A). In successful breeders, EL samples were similar to anoestrus samples, comprising primarily of the phyla *Bacteroidetes* (61.32%), *Firmicutes*, and Proteobacteria. Meanwhile unsuccessful breeder EL samples remained dominated by Proteobacteria (93.32%), along with low abundances of *Bacteroidetes* (3.5%) and *Firmicutes* (2.06%). Successful breeder early lactation samples were highly diverse at the family level, with the most abundant taxa belonging to Muribaculaceae (48.28%), Acidaminococcaceae (13.74%), *Bacteroidaceae* (7.11%), *Desulfovibrionaceae* (4.43%), and Pasteurellaceae (3.39%). In contrast, unsuccessful breeder EL samples were dominated by the family Enterobacteriaceae (82.56%), followed by Pseudomonadaceae (5.46%), Muribaculaceae (3.08%), Pasteurellaceae (3.01%), and Acidaminococcaceae (1.99%) (see Supplementary Tables 5, 6, 7, 8, 9 and 10).

Given these discrepancies in taxonomic composition, we compared taxonomic data between breeding groups using DESeq2 to identify bacterial genera that significantly differed between successful and unsuccessful breeder EL samples. From this data, we observed that successful breeder EL samples contained significantly higher abundances of *Muribaculum* than unsuccessful breeders (Wald P-adj < 0.05; Fig. 7B). Meanwhile unsuccessful breeder EL samples harboured significantly higher abundances of the genera *Pluralibacter*, *Escherichia-Shigella*, *Pseudomonas*, and *Klebsiella* and an unassigned genus from the family Pasteurellaceae (Wald P-adj < 0.05; Fig. 7B) (see Supplementary Table 11).

### Characterisation of the koala pouch microbiota following loss of young

In order to achieve our aim of identifying bacterial taxa associated with loss of young, pouch samples were collected immediately following notification of mortality or upon removal of ill pouch young. As one death occurred outside the deadline for sequencing, a total of six out of seven samples were sequenced for analysis (detailed in Table 2).

**Fig. 8:**
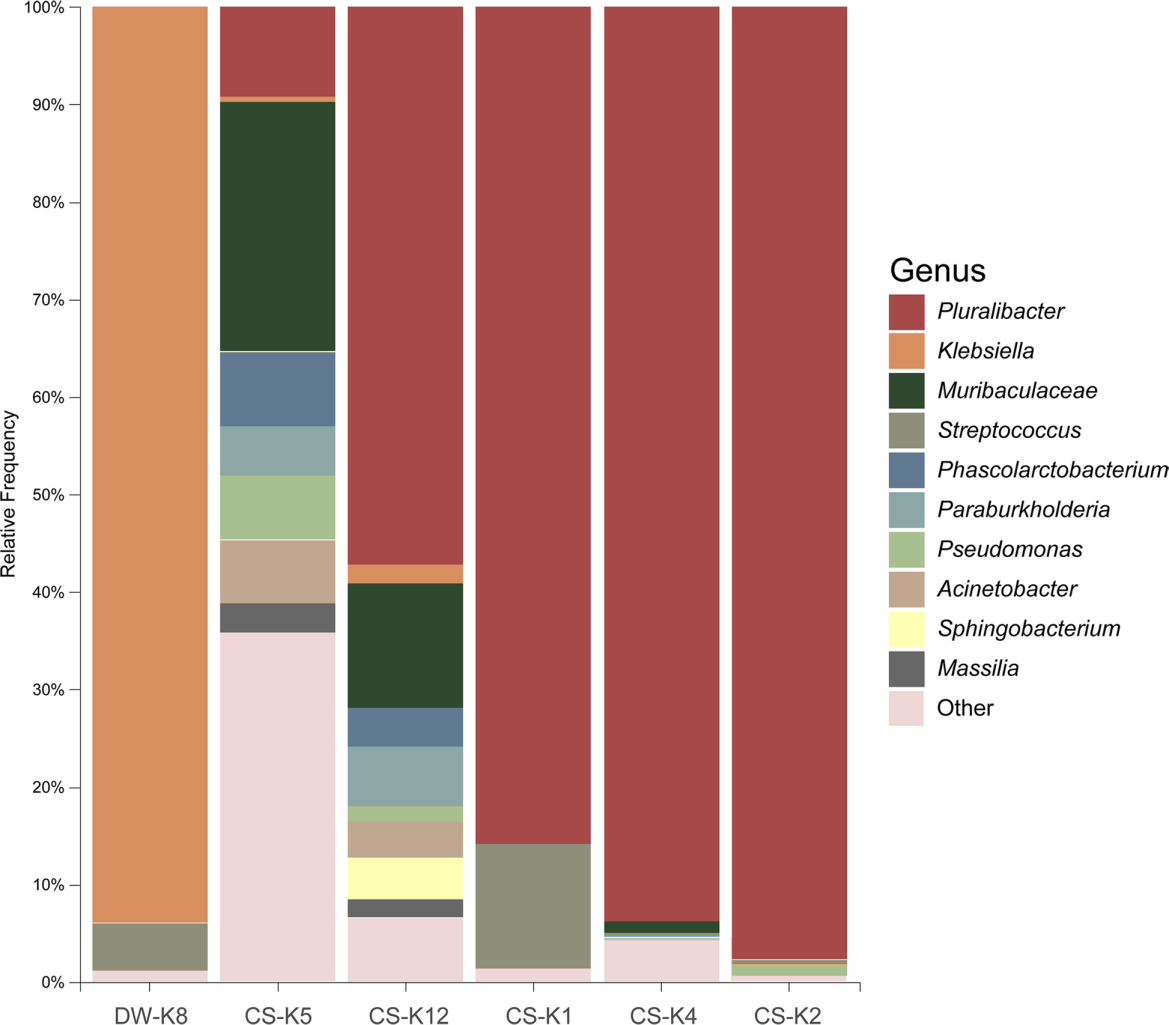
Genus-level bacterial community composition in pouch samples collected following loss of pouch young. The figure displays the relative abundance of bacterial genera in individual loss samples. Visualisation was performed using QIIME2 (v2021.4)

Pouch loss samples were dominated at the phylum level by Proteobacteria (89.93%; range = 49.0–99.3%), followed by *Bacteroidetes* (5.67%) and *Firmicutes* (4.15%; Fig. 8). Enterobacteriaceae represented the most abundant bacterial family overall in loss samples, (84.39%) and was the dominant family in all samples except CS-K5 (Fig. 8). Other families present included Muribaculaceae (3.92%), Streptococcaceae (2.24%), and Moraxellaceae (1.35%).

Of the five animals with *Enterobacteriaceae-*dominated microbiota, all four housed at CWH were dominated by the genus *Pluralibacter* (range = 57.17–97.71% relative abundance), while *Klebsiella* was the dominant genus (93.96% relative abundance) in the pouch of DW-K8 (Fig. 8). Although species-level classification could not be assigned to these taxa using the feature classifier, the closest BLAST hits for their representative sequences were *Pluralibacter gergoviae* (99.56% identity, accession — MT415753) and *Klebsiella pneumoniae* (99.78% identify; accession — CP026153) respectively. Other genera present in these samples with a relative abundance of > 3% included *Muribaculum* (CS- K12 — 12.74%), *Streptococcus* (CS-K1 — 12.77%; DW-K8 — 4.79%), *Sphingobacterium* (CS-K12 – 4.26%), *Phascolartobacterium* (CS-K12 — 3.99%), *Staphylococcus* (CS-K4 — 3.77%), and *Acinetobacter* (CS-K12 — 3.69%) (see Supplementary Table 12).

As detailed in Table 2, the joey of CS-K5 was found deceased in the pouch following suspected head trauma, which occurred after falling from a branch the day prior. The pouch microbiota of CS-K5 at the time of loss exhibited higher taxonomic diversity and less representation of the family Enterobacteriaceae (13.85%) than other loss samples (Fig. 8). The five most abundant genera in the pouch microbiome of this individual were *Muribaculum* (25.59%), *Pluralibacter* (9.2%), *Phascolartobacterium* (7.53%), *Pseudomonas* (6.58%), and *Acinetobacter* (6.49%) (see Supplementary Table 12).

### In vitro* characterisation of Pluralibacter gergoviae and Klebsiella pneumoniae isolates from pouch samples*

To further characterise bacterial strains identified associated with neonatal mortality in our 16S rRNA amplicon sequencing data, suspected *Pluralibacter gergoviae* and *Klebsiella pneumoniae* from pouch samples were revived and identified via API-20E (Table 3).

**Table 3:** *Pluralibacter gergoviae* and *Klebsiella pneumoniae* isolates recovered from pouch samples

Isolate name	Animal	Breeding status	Reproductive time point
CSK1-PG	CS-K1	Unsuccessful	Loss
CSK2-PG	CS-K2	Unsuccessful	Loss
CSK4-PG	CS-K4	Unsuccessful	Loss
CSK12-PG	CS-K12	Unsuccessful	Loss
CSK7-PG	CS-K7	Successful	Very-early lactation
CSK8-PG	CS-K7	Successful	Very-early lactation
DWK3-PG	DW-K3	Successful	Early lactation
DWK23-PG	DW-K23	Unsuccessful	Anoestrus
DWK8-KP	DW-K8	Unsuccessful	Loss

In total, eight *Pluralibacter gergoviae* isolates and one *Klebsiella pneumoniae* isolate were recovered from pouch samples. All *P. gergoviae* isolates exhibited similar macro- and microscopic morphology, presenting as 2–3-mm diameter white, round, and convex colonies on NA under aerobic conditions, which appear smaller (1–2 mm diameter) and translucent under anaerobic conditions (10% CO_2_). *P. gergoviae* isolates were oxidase positive, lactose-fermenting (24 h on MacConkey agar at 37 °C), and presented microscopically as gram-negative straight, singular bacilli, approximately 2 µm long and 0.5 µm wide.

Regarding antibiotic resistance, all 8 *P. gergoviae* isolates were resistant in vitro to ampicillin, 7/8 to tetracycline (1 equivocal), 7/8 to chloramphenicol, 6/8 to augmentin (1 equivocal), and 1/8 to azithromycin (2 equivocals; Table 4). The loss-associated *K. pneumoniae* isolate from DW-K8 was resistant to ampicillin and augmentin, equivocal for azithromycin and tetracycline, and susceptible to chloramphenicol ceftazidime, ciprofloxacin, and gentamycin (Table 4).

**Table 4:** Antibiotic disk-diffusion susceptibility test results

		**Antibiotic**							
		**AMP25**	**TE10**^**a**^	**CIP2.5**	**CN10**^**a**^	**CAZ10**	**AZM15**^**a**^	**C30**	**AMC15**
**ATCC1388**	AR (mm)	0	4	14	9	9.5	4	10	6
	Profile	Resistant	Equivocal	Susceptible	Susceptible	Susceptible	Equivocal^b^	Susceptible	Equivocal^b^
**ATCC33028**	AR (mm)	0	0	12	2.5	9	7	2	4
	Profile	Resistant	Resistant	Susceptible	Resistant	Susceptible	Susceptible	Resistant	Resistant
**CSK1-PG**	AR (mm)	0	0	12	9	9	5	0	3
	Profile	Resistant	Resistant	Susceptible	Susceptible	Susceptible	Susceptible	Resistant	Resistant
**CSK2-PG**	AR (mm)	0	2	14	9	12	6	3	4
	Profile	Resistant	Resistant	Susceptible	Susceptible	Susceptible	Susceptible	Resistant	Resistant
**CSK4-PG**	AR (mm)	0	1	14	9	12	6	5	4
	Profile	Resistant	Resistant	Susceptible	Susceptible	Susceptible	Susceptible	Resistant	Resistant
**CSK12-PG**	AR (mm)	0	0	11	7	9	4	1	1
	Profile	Resistant	Resistant	Susceptible	Susceptible	Susceptible	Equivocal^b^	Resistant	Resistant
**CSK7-PG**	AR (mm)	0	0	14	10	9	5	3	3
	Profile	Resistant	Resistant	Susceptible	Susceptible	Susceptible	Susceptible	Resistant	Resistant
**CSK8-PG**	AR (mm)	0	0	14	9	10	4	1	3
	Profile	Resistant	Resistant	Susceptible	Susceptible	Susceptible	Equivocal^b^	Resistant	Resistant
**DWK2-PG**	AR (mm)	0	4	14	9	12	5	4	6
	Profile	Resistant	Equivocal^b^	Susceptible	Susceptible	Susceptible	Susceptible	Resistant	Equivocal^b^
**DWK23-PG**	AR (mm)	0	0	9	9	7.5	3	0	3
	Profile	Resistant	Resistant	Susceptible	Susceptible	Susceptible	Resistant	Resistant	Resistant
**DWK8-KP**	AR (mm)	0	4	10	8	11	4	9	1
	Profile	Resistant	Equivocal^b^	Susceptible	Susceptible	Susceptible	Equivocal^b^	Susceptible	Resistant

Table 4 provides a summary of antibiotic sensitivity results for each pouch-derived bacterial isolate in addition to *Pluralibacter gergoviae* ATCC33028 and *Klebsiella pneumoniae* ATCC1388, which were used as controls. Sensitivity testing was performed via disk diffusion in adherence to CDS guidelines for *Enterobacteriaceae* [45]. For each isolate, the annular radii (AR) of each antibiotic zone of inhibition is reported in millimetres (mm), followed by the resistance profile inferred from said measurements by CDS guidelines. An *AR* > 6 mm indicates resistance to antibiotics unless marked with ^a^, which have AR cutoffs of > 4 mm

^b^Equivocal profiles were determined where the *AR* = cutoff and are generally referred for further testing where possible. *AMP25*, ampicillin (25 µg); *TE10*, tetracycline (10 µg); *CIP2.5*, ciprofloxacin (2.5 µg); *CN10*, gentamycin (10 µg); *CAZ10*, ceftazidime (10 µg); *AZM15*, azithromycin (15 µg); *C30*, chloramphenicol (30 µg); *AMC15*, augmentin (15 µg)

## Discussion

### Changes in koala pouch microbiota diversity during development are reflective of marsupial-specific adaptation to ex utero development

Koala neonates are exposed to the external environment after birth without a functioning immune system and are thus vulnerable to infection from environmental microorganisms. Several maternal host-defence strategies are understood to offer antimicrobial protection to marsupial young in the pouch; however, their effect on the pouch microbiota remains poorly understood [15]. As such, the first aim of this study was to investigate how bacterial communities in the koala pouch changed across the reproductive cycle.

We found that bacterial diversity in the pouch changed across the reproductive cycle, characterised by a significant reduction in alpha diversity immediately following parturition. These changes in the koala pouch microbiota are consistent with cultivation-dependent observations in other marsupials such as the quokka, tammar wallaby, and brushtail possum, where bacterial clearance from the pouch was observed immediately post-partum [16, 18–20]. Our observations in the koala are also highly comparable to those made by Weiss et al. (2021), in their study of southern hairy-nosed wombat (SHNW) pouches, where microbial diversity was significantly lower in lactating wombats versus non-cycling animals [23].

In healthy koala pouches, significant reductions in bacterial diversity towards parturition corresponded with a compositional shift from a profile co-dominated by the phyla, *Bacteroidetes*, Proteobacteria, and *Firmicutes*, to one dominated by Proteobacteria, and represented primarily by gram-negative *Enterobacteriaceae* and *Pseudomonadaceae*. Interestingly, this is taxonomically similar to the brushtail possum pouch, which contained *Enterobacteriaceae*, *Pseudomonadaceae*, and *Alcaligenaceae* following parturition, but differs from wallaby, wombat, and quokka pouches — all of which were found to be dominated by gram-positive *Corynebacterium* [16, 18–20, 23]. One explanation for these differences is that unlike ground-dwelling quokka, wombat, and wallaby pouches, which are exposed to soil and ground cover rich in *Actinobacteria*, the pouches of arboreal koalas and possums are more frequently in contact with tree bark and foliage, which are commonly dominated by Proteobacteria [46]. Another explanation is that *Enterobacteriaceae*, which are highly abundant in the koala rectal microbiota, are translocated from the cloaca and into the pouch via migrating neonates [47, 48]. However, future comparative work with paired sampling would be required to elucidate interspecies differences in taxonomic composition.

### Comparison of pouch microbiota between successful and unsuccessful breeding koalas reveals a dysbiotic compositional profile associated with loss of pouch young

To identify any longitudinal changes in the pouch microbiota associated with loss of pouch young in captive koalas, we compared the microbiota of koalas with successful breeding outcomes to those who went on to lose young across the maternal reproductive cycle. When stratified by breeding group, we identified a number of key differences in pouch microbiota composition between successful and unsuccessful breeders. Firstly, we observed that while pouches of both breeding groups contained similar distributions of the phyla *Bacteroidetes*, Proteobacteria, and *Firmicutes* prior to mating, unsuccessful breeder pouches were dominated by *Enterobacteriaceae*, which comprised < 1% in successful breeders during the same period. Similarly, despite reduced abundances of *Bacteroidetes* and *Firmicutes* occurring in both groups following parturition, the relative abundance of *Enterobacteriaceae*, represented primarily by the genus *Pluralibacter*, was higher in unsuccessful breeders than successful breeders.

The most striking difference, however, was observed in the months following parturition during early lactation (EL). Compared to successful breeders, whose microbiota returned to a *Bacteroidetes*-dominant compositional profile similar to pre-mating, unsuccessful breeder pouches remained dominated by *Enterobacteriaceae* and exhibited significantly increased abundances of *Pluralibacter*, *Escherichia-Shigella*, *Klebsiella*, and *Pseudomonas*. Unsuccessful breeder pouches also contained low abundances of Muribaculaceae, Acidaminococcaceae, and *Bacteroidaceae*, which dominated successful breeder pouches and contain several functionally important taxa identified in the gastrointestinal (GI) microbiome of adult and juvenile captive koalas [49–51]. We then observed this *Enterobacteriaceae-*dominant, low diversity pouch microbiota profile to persist in unsuccessful breeder pouches until loss of young occurred, with five out of six mortality-associated samples dominated by either *Pluralibacter* or *Klebsiella*. This is consistent with previous cultivation-based observations in the koala [12], where continued growth of pathogenic taxa including *Klebsiella* spp. from pouch samples during early development only occurred in females who later lost young. However, while those findings led to the conclusion that healthy koala pouches must be sterile, our results demonstrate that unsuccessful breeder pouches instead represent a deviation from a community profile dominated by highly fastidious, difficult to culture anaerobes, rather than sterility.

Based on these compositional differences, we propose that sustained overgrowth of *Enterobacteriaceae* and the loss of functionally important taxa in the pouch microbiota reflect a persistent dysbiosis event occurring in unsuccessful individuals. Similar patterns of *Enterobacteriaceae* overgrowth or ‘blooms’ are the most common driver of dysbiosis in the mammalian GI microbiome, especially during periods of local inflammation [52, 53]. While it is unknown whether dysbiosis in the koala pouch arises from the neonatal GI tract or the external environment, similar overgrowth of *Enterobacteriaceae* in the pouch environment could have life-threatening consequences for developing young. For instance, induction of a high-O_2_, pro-inflammatory environment in the pouch due to overabundant immunogenic *Enterobacteriaceae* could result in the loss of anaerobic endosymbionts and thus altered community function [54, 55]. This might explain the lower abundances of Muribaculaceae and Acidaminococcaceae in the pouch microbiota of unsuccessful koalas compared to healthy koalas, though further research would be required to determine their functional role in pouch development. The increased selective pressure resulting from inflammation could also increase virulence expression in taxa such as *P. gergoviae* and *K. pneumoniae*, increasing the likelihood and severity of neonatal infection due to enhanced invasion, motility, immune evasion, and nutrient acquisition capabilities [52–57]. Although further characterisation of maternal immune responses to opportunistic pathogens such as *K. pneumoniae* and *P. gergoviae* is required to test these assumptions, exposure to large populations of virulent *Enterobacteriaceae* has been implicated with neonatal bacteraemia and septicaemia in eutherian neonates, which typically have greater immune function than marsupial neonates [58–61]. As such, prolonged exposure in highly altricial koala neonates is likely a considerable source of mortality during early development.

One avenue of future research is the characterisation of taxa from the family Muribaculaceae which dominated the pouch microbiota of successful breeders during most of the reproductive cycle and formed an almost inverse relationship with *Enterobacteriaceae* over time. Muribaculaceae (phylum — *Bacteroidetes*) are a largely uncultured family of obligate anaerobic bacteria commonly found at mammalian gastrointestinal mucosa, where they feed primarily on complex carbohydrates such as host, plant, and alpha glycans [62]. In addition to our observations in the koala pouch, Muribaculaceae have also been identified at varying abundances in the koala oral, cloacal, ocular, and urogenital tract microbiome of both wild and captive koalas [47, 48]. In particular, several unidentified genera/species of Muribaculaceae have been found abundantly in the koala GI microbiome of both adults and juveniles, which might play a specialised role in digestion and metabolism of *Eucalyptus* [50, 51]*.* Interestingly, however, a recent study of the developing koala faecal microbiome by Blyton et al. identified Muribaculaceae at an average abundance of 11.34% in pre-papping joey faeces, which is comparatively lower than the 48.27% abundance observed in successful breeder pouches during early lactation [51]. This leads to the questions of whether Muribaculaceae in the pouch are distinct from those in the GI tract and if they play a unique protective or functional role beneficial for developing young. As *Muribaculum intestinale* has been found to play a protective immunomodulatory role in the IgA neutralisation of *Helicobacter pylori* in mice [63], elucidating similar protective mechanisms by Muribaculaceae in the koala pouch could prove extremely useful in understanding the functional role of microbes in the koala pouch.

### Implications for captive koala husbandry and conservation

Bacterial infections represent a common risk associated with long-term captivity, as increased contact with humans and other species exposes animals to foreign microorganisms not present within their natural habitat [64]. Prior reports have identified bacterial infection as a frequent cause of neonatal mortality amongst captive koala colonies with current captive husbandry guidelines listing several taxa associated with mortality [11, 12, 65]. While we identified one of these organisms, *K. pneumoniae*, in association with mortality in a single individual, the majority of cases in our study were associated with *P. gergoviae*, which is not listed in these guidelines but has been previously identified in necropsy reports provided by veterinarians [66]. *P. gergoviae* (formerly *Enterobacter gergoviae*) is a gram-negative, facultative anaerobe bacillus from the family Enterobacteriaceae found in a wide array of diverse environments, where it is best known as a common contaminant of cosmetic products due to extensive paraben and biocide resistance [67, 68]. *P. gergoviae* is an opportunistic pathogen of humans linked to a wide array of nosocomial and community-acquired infections and is often responsible for fatal neonatal sepsis outbreaks in developing countries [58, 59, 69–71]. Unlike *K. pneumoniae*, *P. gergoviae* has not previously been detected in other regional microbiota in the koala and thus may represent an exogenously acquired organism rather than normal regional flora. Our study also failed to identify *P. gergoviae* amongst any environmental samples from *Eucalyptus* feed, poles, or other enclosure surfaces, meaning we cannot determine any environmental reservoirs. However, as all *P. gergoviae* strains recovered during our study exhibited resistance to most commonly used antimicrobials in the treatment of koalas, it likely to have derived from a veterinary or clinical environment.

As endangered koala populations continue to decline at their northern distribution, captive breeding programmes have become an essential component of species conservation. Managing and breeding koalas in captivity, however, can present several unique challenges such as variable conception rates and high neonatal mortality rates, which often place major strains on breeding outputs. While pregnancy outcomes in captive koalas have been greatly improved through recent advances in artificial insemination and in vitro fertilisation technology, the issue of neonatal mortality has remained largely unaddressed over the past three decades. This is despite pouch young mortality contributing to a 10–30% reduction in breeding output on average and annual reductions exceeding 50% [11–13]. Though further-reaching investigations are required to understand neonatal mortality in the wider captive population, the results of this study highlight the need for more transparent and robust methods of reporting neonatal mortality amongst koala conservation facilities. Due to the presence of multiple potential causative agents, improved monitoring should also be implemented through routine post-mortem examinations of deceased young, cultivation of pouch swabs, and antimicrobial susceptibility testing of mortality-associated microorganisms. By collecting such data, we can then begin to develop preventative screening tools and treatment protocols to improve breeding outcomes in future.

## Conclusions

Overall, this study provides the first cultivation-independent evidence to support the hypothesis that perturbations in the marsupial pouch microbiota influence reproductive outcomes. Specifically, our results demonstrate that dysbiosis in the pouch microbiota of captive koalas is associated with neonatal mortality. This dysbiosis was characterised primarily by persistent dominance of *Enterobacteriacae* across the reproductive cycle as well as significantly reduced abundances of Muribaculaceae and was distinct from the largely Muribaculaceae-dominant microbiota of successful breeders. In particular, we identified two species, *Klebsiella pneumoniae* and *Pluralibacter gergoviae*, which were present at very high abundances prior to, and immediately following mortality, and are thus implicated with mortality. Although *K. pneumoniae* has been previously identified in association with mortality [11, 12], this study represents the first published account of multidrug-resistant *P. gergoviae* associated with disease in koalas. This is important, as it suggests the possible emergence of new pathogenic taxa associated with neonatal mortality in the nearly three decades since this issue was last mentioned in the literature. Based on these findings, we emphasise that more transparent record sharing is required to determine the full extent of this issue, and that improved monitoring of emerging pathogens may prevent the occurrence of larger outbreaks. We also suggest that conservation facilities, veterinarians, and researchers work collaboratively to develop preventative diagnostics for at-risk animals and standardised treatment protocols, as well as potential novel probiotics for long-term prevention. Furthermore, this study highlights the need for further research into microbial communities of the mammalian female reproductive system, which remain a highly understudied, yet important facet of overall reproductive health.

## Supplementary Information

**Additional file 1: SI** **Table 1.** Sample metadata. **SI Table 2.** Summary of bacterial culture results from longitudinally sampled koala pouch samples. **SI Table 3.** Summed relative abundance of bacterial phyla per time point (all animals). **SI Table 4.** Relative abundance of bacterial families per time point (all animals). **SI Table 5.** Relative abundance of bacterial phyla in successful breeder pouches stratified by reproductive time point. **SI Table 6.** Relative abundance of bacterial phyla in unsuccessful breeder pouches stratified by reproductive time point. **SI Table 7.** Relative abundance of bacterial families in successful breeder pouches stratified reproductive time point. **SI Table 8.** Relative abundance of bacterial families in unsuccessful breeder pouches stratified by reproductive time point. **SI Table 9.** Summed relative abundance of bacterial genera in successful breeder pouches stratified by reproductive time point. **SI Table 10.** Summed relative abundance of bacterial genera in unsuccessful breeder pouches stratified by reproductive time point. **SI Table 11.** Differentially abundant bacterial genera between successful and unsuccessful breeder pouch samples obtained during early lactation. **SI Table 12.** Relative abundance of bacterial genera in pouch samples following loss of pouch young.

## Data Availability

Demultiplexed sequences from this study are available from the NCBI Short Read Archive (SRA) repository under BioProject PRJNA903532. QIIME2 artefacts and analysis scripts can be found at https://github.com/Tibieuan/koala_pouch_microbiome.

## Abbreviations

AGRF: Australian Genome Research Facility
AMP: Antimicrobial peptide(s)
AN: Anoestrus
ANOSIM: Analysis of similarity
API: Analytical profile index
AR: Annular radii
AST: Antibiotic susceptibility testing
ASV: Amplicon sequence variant
ATCC: American Tssue Culture Collection
BG: Breeding group
BHI: Brain-heart infusion
BLAST: Basic local alignment tool
Bp: Base pair(s)
Cat. no.: Catalogue number
CDS: Calibrated Dichotomous Sensitivity Test
CO2: Carbon dioxide
CWH: Currumbin Wildlife Hospital
ddH2O: Double-distilled water
DNA: Deoxyribonucleic acid
DW: Dreamworld
EL: Early lactation
EDTA: Ethylenediaminetetraacetic acid
KoRV: Koala retrovirus
KP: *Klebsiella* *pneumoniae*
NA: Nutrient agar
NaCl: Sodium chloride
NCBI: National Centre for Biomedical Innovation
NCTC: National Centre for Tissue Culture
Nr: Non-redundant
O_2_: Oxygen
OD600: Optical density at 600 nm
PCoA: Principal coordinates analysis
PE: Post emergence
PERMANOVA: Permutational analysis of variance
pH: Power of hydrogen
QIIME: Quantitative insights into microbial ecology
QUT: Queensland University of Technology
rRNA: Ribosomal ribonucleic acid
SHNW: Southern hairy-nosed wombat
USA: United States of America
VEL: Very-early lactation

## References

[1] Gonzalez-Astudillo V, Allavena R, McKinnon A, Larkin R, Henning J (2017). Decline causes of koalas in South East Queensland, Australia: a 17-year retrospective study of mortality and morbidity”. Sci Reports..

[2] Adams-Hosking C, McBride MF, Baxter G, Burgman M, de Villiers D, Kavanagh R (2016). Use of expert knowledge to elicit population trends for the koala (Phascolarctos cinereus). Biodivers Res.

[3] McAlpine C, Lunney D, Melzer A, Menkhorst P, Phillips S, Phalen D (2015). Conserving koalas: a review of the contrasting regional trends, outlooks and policy challenges. Biol Conserv..

[4] Nyari S, Waugh CA, Dong J, Quigley BL, Hanger J, Loader J (2017). Epidemiology of chlamydial infection and disease in a free-ranging koala (Phascolarctos cinereus) population. PLoS ONE.

[5] McCallum H, Kerlin DH, Ellis W, Carrick F (2018). Assessing the significance of endemic disease in conservation—koalas, chlamydia, and koala retrovirus as a case study. Conserv Letters..

[6] Davies N, Gramotnev G, Seabrook L, McAlpine C, Baxter G, Lunney D (2014). Climate-driven changes in diet composition and physiological stress in an arboreal folivore at the semi-arid edge of its distribution. Biol Conserv..

[7] Gentle M, Allen BL, Oakey J, Speed J, Harriott L, Loader J (2019). Genetic sampling identifies canid predators of koalas (Phascolarctos cinereus) in peri-urban areas. Landscape and Urban Planning..

[8] ND Eeden LM, Mahony M, Herman K, DJ. Ehmke G, O’Connor J, Bino G, Taylor M, D CR, Impacts of the unprecedented 2019–2020 bushfires on Australian animals.Ultimo, NSW, Australia 2020.

[9] Seddon JM, Schultz B (2020). Koala conservation in Queensland, Australia: a role for assisted gene flow for genetic rescue?. Conservation Genetics in Mammals: Integrative Research Using Novel Approaches, J. Ortega and J. E. Maldonado, Eds., ed.

[10] Tobey JR, Andrus CH, Doyle L, Thompson VD, Bercovitch FB (2006). Maternal effort and joey growth in koalas (Phascolarctos cinereus). J Zool.

[11] O'Callaghan PG (1996). Growth and mortality of koala pouch and back young, in Australian Koala Foundation Conference on the Status of the Koala, Currumbin, Queensland, Australia.

[12] Osawa R, Blanshard WH, O'Callaghan PG (1992). Microflora of the pouch of the koala (Phascolarctos cinereus). J Wildl Dis.

[13] Blanshard WH (1994). Medicine and husbandry - koalas,” in Post Graduate Committee in Veterinary Science, The University of Sydney.

[14] Edwards MJ, Deakin JE (2013). The marsupial pouch: implications for reproductive success and mammalian evolution. Aust J Zool.

[15] Cheng Y, Belov K. Antimicrobial protection of marsupial pouch young. Front Microbiol. 2017;8:354. 10.3389/fmicb.2017.00354 PMC5339227 28326070

[16] Chhour K-L, Hinds LA, Jacques NA, Deane EM (2010). An observational study of the microbiome of the maternal pouch and saliva of the tammar wallaby, Macropus eugenii, and of the gastrointestinal tract of the pouch young. Microbiology.

[17] Cheng Y, Fox S, Pemberton D, Hogg C, Papenfuss AT, Belov K (2015). The Tasmanian devil microbiome—implications for conservation and management. Microbiome..

[18] Deakin JE, Cooper DW (2004). Characterisation of and immunity to the aerobic bacteria found in the pouch of the brushtail possum Trichosurus vulpecula. Comp Immunol Microbiol Infect Dis..

[19] Old JM, Deane EM (1998). The effect of oestrus and the presence of pouch young on aerobic bacteria isolated from the pouch of the tammar wallaby, Macropus eugenii. Comp Immunol Microbiol Infect Dis..

[20] Charlick J, Manessis C, Stanley N, Waring H, Cockson A (1981). Quantitative alterations of the aerobic bacterial flora of the pouch of Setonix brachyurus (Quokka) during oestrus, anoestrus, pregnancy and lactating anoestrus (pouch young). Aust J Exp Biol Med Sci..

[21] Stannard HJ, Miller RD, Old JM (2020). Marsupial and monotreme milk—a review of its nutrient and immune properties. Peer J..

[22] Morris KM, O’Meally D, Zaw T, Song X, Gillett A, Molloy MP (2016). Characterisation of the immune compounds in koala milk using a combined transcriptomic and proteomic approach. Scientific Reports..

[23] Weiss S, Taggart D, Smith I, Helgen KM, Eisenhofer R (2021). Host reproductive cycle influences the pouch microbiota of wild southern hairy-nosed wombats (Lasiorhinus latifrons). Animal Microbiome..

[24] Peel E, Cheng Y, Djordjevic JT, Fox S, Sorrell TC, Belov K (2016). Cathelicidins in the Tasmanian devil (Sarcophilus harrisii). Scientific Reports.

[25] Moreno I, Codoñer FM, Vilella F, Valbuena D, Martinez-Blanch JF, Jimenez-Almazán J (2016). Evidence that the endometrial microbiota has an effect on implantation success or failure. Am J Obstet Gynecol.

[26] van de Wijgert JHHM (2017). The vaginal microbiome and sexually transmitted infections are interlinked: consequences for treatment and prevention. PLoS Med.

[27] Al-Memar M, Bobdiwala S, Fourie H, Mannino R, Lee YS, Smith A (2020). The association between vaginal bacterial composition and miscarriage: a nested case-control study. Braz J Gynecol.

[28] Heil BA, Paccamonti DL, Sones JL (2019). Role for the mammalian female reproductive tract microbiome in pregnancy outcomes. Physiol Genomics..

[29] Cherne MD, Cole AL, Newberry L, Schmidt-Owens M, Deichen M, Cole AM (2020). Matrix metalloproteinases expressed in response to bacterial vaginosis disrupt the endocervical epithelium, increasing transmigration of HIV. Infect Immun.

[30] Nardini P, Ñahui Palomino RA, Parolin C, Laghi L, Foschi C, Cevenini R (2016). Lactobacillus crispatus inhibits the infectivity of Chlamydia trachomatis elementary bodies, in vitro study. Sci Reports..

[31] West AG, Waite DW, Deines P, Bourne DG, Digby A, McKenzie VJ (2019). The microbiome in threatened species conservation. Biol Conserv..

[32] Eisenhofer R, Minich JJ, Marotz C, Cooper A, Knight R, Weyrich LS (2019). Contamination in low microbial biomass microbiome studies: issues and recommendations. Trends Microbiol..

[33] Bolyen E, Rideout JR, Dillon MR, Bokulich NA, Abnet CC, Al-Ghalith GA (2019). Reproducible, interactive, scalable and extensible microbiome data science using QIIME 2. Nature Biotechnol..

[34] Amir A, McDonald D, Navas-Molina JA, Kopylova E, Morton JT, Zech Xu Z (2017). Deblur rapidly resolves single-nucleotide community sequence patterns. mSystems..

[35] Price MN, Dehal PS, Arkin AP (2010). FastTree 2 – approximately maximum-likelihood trees for large alignments. PLoS ONE.

[36] Katoh K, Standley DM (2013). MAFFT multiple sequence alignment software version 7: improvements in performance and usability. Mol Biol Evol.

[37] Quast C, Pruesse E, Yilmaz P, Gerken J, Schweer T, Yarza P (2013). The SILVA ribosomal RNA gene database project: improved data processing and web-based tools. Nucleic Acids Res.

[38] Robeson MS, O'Rourke DR, Kaehler BD, Ziemski M, Dillon MR, Foster JT (2021). RESCRIPt: reproducible sequence taxonomy reference database management. PLoS Comput Biol.

[39] Pedregosa F, Varoquaux G, Gramfort A, Michel V, Thirion B, Grisel O (2011). Scikit-learn: machine learning in Python. J Mach Learn Res.

[40] McMurdie PJ, Holmes S (2013). phyloseq: an R package for reproducible interactive analysis and graphics of microbiome census data. PLoS ONE.

[41] Davis NM, Proctor DM, Holmes SP, Relman DA, Callahan BJ (2018). Simple statistical identification and removal of contaminant sequences in marker-gene and metagenomics data. Microbiome..

[42] Wickham H (2009). ggplot2: elegant graphics for data analysis.

[43] Shannon CE (1948). A mathematical theory of communication. Bell Syst Tech J..

[44] Love MI, Huber W, Anders S (2014). Moderated estimation of fold change and dispersion for RNA-seq data with DESeq2. Genome Biology..

[45] Bell JNPSM, Rafferty DL, Allerton JK, James PM. Antibiotic susceptibility testing by the CDS method: a manual for medical and veterinary laboratories. Available: http://cdstest.net/wordpress/wp-content/uploads/10th-Edition-Modified-October-2021.pdf. 2019.

[46] Varela C, Sundstrom J, Cuijvers K, Jiranek V, Borneman A (2020). Discovering the indigenous microbial communities associated with the natural fermentation of sap from the cider gum Eucalyptus gunnii. Scientific Reports..

[47] Alfano N, Courtiol A, Vielgrader H, Timms P, Roca AL, Greenwood ADJSr (2015). Variation in koala microbiomes within and between individuals: effect of body region and captivity status. Scientific Reports..

[48] Vidgen ME, Hanger J, Timms P (2017). Microbiota composition of the koala (Phascolarctos cinereus) ocular and urogenital sites, and their association with Chlamydia infection and disease. Scientific Reports..

[49] Brice KL, Trivedi P, Jeffries TC, Blyton MDJ, Mitchell C, Singh BK, Moore BD. “The Koala (Phascolarctos cinereus) faecal microbiome differs with diet in a wild population. PeerJ. 2019;7:e6534. 10.7717/peerj.6534 PMC6448554 30972242

[50] Shiffman ME, Soo RM, Dennis PG, Morrison M, Tyson GW, Hugenholtz P (2017). Gene and genome-centric analyses of koala and wombat fecal microbiomes point to metabolic specialization for Eucalyptus digestion. PeerJ..

[51] Blyton MDJ, Soo RM, Hugenholtz P, Moore BD (2022). Maternal inheritance of the koala gut microbiome and its compositional and functional maturation during juvenile development. Environ Microbiol..

[52] Baldelli V, Scaldaferri F, Putignani L, Del Chierico F (2021). The role of Enterobacteriaceae in gut microbiota dysbiosis in inflammatory bowel diseases. Microorganisms.

[53] Shin N-R, Whon TW, Bae J-W (2015). Proteobacteria: microbial signature of dysbiosis in gut microbiota. Trends Biotechnol..

[54] Zeng MY, Inohara N, Nuñez G (2017). Mechanisms of inflammation-driven bacterial dysbiosis in the gut. Mucosal Immunol.

[55] Petersen C, Round JL (2014). Defining dysbiosis and its influence on host immunity and disease. Cell Microbiology.

[56] Messer JS, Chang EB. Chapter 36 - Microbial physiology of the digestive tract and its role in inflammatory bowel diseases,” in physiology of the gastrointestinal tract (Sixth Edition), H. M. Said, Ed., ed. London: Academic Press; 2018. pp. 795–810.

[57] Wood TE, Aksoy E, Hachani A (2020). From welfare to warfare: the arbitration of host-microbiota interplay by the type VI secretion system. Front Cell Infect Microbiol..

[58] Ganeswire R, Thong KL, Puthucheary SD (2003). Nosocomial outbreak of Enterobacter gergoviae bacteraemia in a neonatal intensive care unit. J Hospital Infect..

[59] Boban N, Jerončić A, Punda-Polić V (2011). Outbreak of nosocomial bacteremias, caused by Enterobacter gergoviae and Enterobacter aerogenes, in the neonatal intensive care unit, case - control study. Signa Vitae.

[60] Gray J, Arvelo W, McCracken J, Lopez B, Lessa FC, Kitchel B (2012). An outbreak of Klebsiella pneumoniae late-onset sepsis in a neonatal intensive care unit in Guatemala. Am J Infect Control..

[61] Wisgrill L, Lepuschitz S, Blaschitz M, Rittenschober-Böhm J, Diab-El Schahawi M, Schubert S (2019). Outbreak of yersiniabactin-producing Klebsiella pneumoniae in a neonatal intensive care unit. Pediatr Infect Dis J..

[62] Lagkouvardos I, Lesker TR, Hitch TCA, Gálvez EJC, Smit N, Neuhaus K (2019). Sequence and cultivation study of Muribaculaceae reveals novel species, host preference, and functional potential of this yet undescribed family. Microbiome..

[63] Satoh-Takayama N, Kato T, Motomura Y, Kageyama T, Taguchi-Atarashi N, Kinoshita-Daitoku R (2020). Bacteria-induced group 2 innate lymphoid cells in the stomach provide immune protection through induction of IgA. Immunity..

[64] Ossiboff RJ, Origgi FC, Stacy NI (2020). Editorial: health and disease in free-ranging and captive wildlife. Front Vet Sci..

[65] Jackson S, Perry L, O'Callaghan P, Spittal D, Romer L, Reid K. Koala (Phascolarctos cinereus) - captive husbandry guidelines. Australasian Zookeeping, 2000. URL: http://www.australasianzookeeping.org/Husbandry%20Manuals/KOALA%20Husbandry%20Manual.pdf.

[66] Nicolson VB, Barnes M. Personal communication of veterinary records detailing neonatal mortality in koalas due to bacterial infection. unpublished, 2020.

[67] Cunningham-Oakes E, Pointon T, Murphy B, Connor TR, Mahenthiralingam E (2020). Genome sequence of Pluralibacter gergoviae ECO77, a multireplicon isolate of industrial origin. Microbiol Resour Announcements.

[68] Chan K-G, Tee KK, Yin W-F, Tan J-Y (2014). Complete genome sequence of Pluralibacter gergoviae FB2, an N-acyl homoserine lactone-degrading strain isolated from packed fish paste. Microbiol Resour Announcements.

[69] Khashei R, EdalatiSarvestani F, Malekzadegan Y, Motamedifar M (2020). The first report of Enterobacter gergoviae carrying bla (NDM-1) in Iran. Iran J Basic Med Sci..

[70] Freire MP, de Oliveira Garcia D, Cury AP, Spadão F, Di Gioia TSR, Francisco GR (2016). Outbreak of IMP-producing carbapenem-resistant Enterobacter gergoviae among kidney transplant recipients. J Antimicrob Chemother..

[71] Kesieme E, Kesieme C, Akpede G, Okonta K, Dongo A, Adewuyi G (2012). Tension pneumatocele due to Enterobacter gergoviae pneumonia: a case report. Case Reports Med..

